# Awareness, Knowledge, Attitude, and Acceptance of Dental Implants Among the Population of North Maharashtra: A Cross-Sectional Questionnaire-Based Study

**DOI:** 10.7759/cureus.110700

**Published:** 2026-06-11

**Authors:** Rushikesh Wayal, Priyadarshani Pawar, Girija Dodamani, Arun Dodamani, Bhavna Bhairi, Vaishnavi Sarda

**Affiliations:** 1 Prosthodontics, Jawahar Medical Foundation's Annasaheb Chudaman Patil Memorial Dental College, Dhule, IND; 2 Public Health Dentistry, Jawahar Medical Foundation's Annasaheb Chudaman Patil Memorial Dental College, Dhule, IND

**Keywords:** attitude, awareness, cross-sectional studies, dental implants, knowledge

## Abstract

Introduction: Dental implants have revolutionized the rehabilitation of missing teeth by improving aesthetics, function, and long-term stability. Public awareness and acceptance of implant therapy are essential for a successful treatment. The present study aimed to assess the awareness, knowledge, attitude, and acceptance of dental implants among the population of North Maharashtra and to evaluate their association with demographic variables, such as gender, education, and monthly family income.

Materials and methods: A descriptive cross-sectional questionnaire-based survey was conducted with 276 participants recruited from prosthodontics outpatient departments and community outreach programs at the Jawahar Medical Foundation’s Annasaheb Chudaman Patil Memorial Dental College, Dhule. A structured questionnaire, consisting of demographic details and 20 implant-related questions, was administered. The questionnaire evaluated four domains: basic awareness, specific knowledge, attitude, willingness, and barriers to information sources. Statistical analysis was performed using SPSS version 26.0 (released 2018; IBM Corp., Armonk, USA). Independent t-test, one-way analysis of variance (ANOVA), Tukey's post-hoc test, and Pearson's correlation analysis were used, with p < 0.05 considered statistically significant.

Results: Of the participants, 152 (55.1%) were males, and 124 (44.9%) were females. Females demonstrated significantly higher total questionnaire scores (10.64 ± 4.10) than males (9.55 ± 4.35) (p = 0.037). Educational status had a strong positive association with implant awareness and acceptance. Participants with postgraduate education demonstrated significantly higher total scores (13.55 ± 3.80) compared to participants without formal education (6.40 ± 3.20) (p < 0.001). Similarly, participants belonging to the highest monthly income group (>₹50,000) exhibited greater total scores (13.20 ± 3.70) than the lowest income group (<₹10,000) (7.55 ± 3.50) (p < 0.001). Significant positive correlations were found between basic awareness and specific knowledge (r = 0.58), attitude and specific knowledge (r = 0.62), and attitude with basic awareness (r = 0.49). All p < 0.001, indicating that enhanced awareness improves knowledge and willingness for dental implants.

Conclusion: The present study demonstrated moderate awareness of dental implants among the population of North Maharashtra. Higher educational and socioeconomic status was significantly associated with improved awareness, knowledge, and acceptance of implant therapy. Public education initiatives and enhanced patient counseling are essential to improve awareness and accessibility of dental implants.

## Introduction

Replacement of missing teeth is essential for maintaining oral function, esthetics, phonetics, and psychological well-being. Tooth loss can adversely affect mastication, speech, facial appearance, and quality of life, thereby influencing social interactions and self-confidence [1]. Traditionally, removable and fixed partial dentures have been widely used for rehabilitation. However, dental implants have emerged as a predictable and long-term treatment modality for replacing missing teeth [2]. Dental implants provide superior stability, preservation of alveolar bone, improved chewing efficiency, and enhanced patient satisfaction compared to conventional prosthetic options [3,4]. Despite these advantages, the acceptance and utilization of implant therapy among the general population remains influenced by several socioeconomic and educational factors [2].

Awareness and knowledge of dental implants play major roles in treatment acceptance. Patients with adequate information about implant placement, longevity, maintenance, and benefits are more likely to choose implants as a treatment option [5]. Conversely, misconceptions, fear of surgery, high treatment costs, and lack of reliable information may limit patients’ willingness to undergo implant therapy [6]. Previous studies conducted in different regions of India have demonstrated varying levels of awareness and acceptance of dental implants among the public, indicating the influence of literacy, income, and accessibility of dental services on patient perception [5,7]. However, limited literature is available regarding implant awareness among the population of North Maharashtra, where socioeconomic diversity and differences in educational status may significantly affect the public understanding of advanced dental treatment modalities.

The assessment of public awareness and attitudes toward dental implants is important for identifying knowledge gaps and planning targeted educational interventions. Understanding the barriers preventing patients from opting for implant therapy may also help dental professionals improve patient counseling and treatment acceptance. This study aimed to assess the awareness, knowledge, attitude, and acceptance of dental implants among the population of North Maharashtra. The objectives were to evaluate the level of basic awareness and specific knowledge regarding dental implants, assess patient attitudes and willingness toward implant therapy, identify major barriers and sources of information related to implants, and determine the association between implant awareness scores and demographic variables, such as gender, education, and monthly family income.

## Materials and methods

Study design and setting

The present study was designed as a descriptive cross-sectional questionnaire-based survey conducted at Jawahar Medical Foundation’s Annasaheb Chudaman Patil Memorial Dental College, Dhule. The study was conducted in prosthodontic outpatient departments and community outreach programs to obtain responses from individuals belonging to various educational and socioeconomic backgrounds across North Maharashtra. The study was conducted over a period of four months from January 2026 to April 2026. The study duration included questionnaire preparation, pilot testing, participant recruitment, data collection, and statistical analyses.

Ethical approval and informed consent

Before the commencement of the study, ethical clearance was obtained from the Institutional Ethics Committee (EC/NEW/INST/2022/2059/SS207 dated 27.12.25). The study was conducted in accordance with the ethical principles of the Declaration of Helsinki. All participants were informed about the objectives and methodology of the study, and written informed consent was obtained before enrolment. Confidentiality and anonymity of participant responses were maintained throughout the study.

Eligibility criteria

The study included individuals aged 18 years and above who were residents of North Maharashtra and were willing to participate in the study. Participants were required to understand and respond to the questionnaire independently. Dental professionals and dental students were excluded to avoid professional knowledge bias. Individuals with cognitive or communication difficulties affecting questionnaire completion, those unwilling to participate, and those who submitted incomplete questionnaires were also excluded from the study.

Sample size estimation

The sample size was estimated using G*Power software version 3.1 (Heinrich-Heine-Universität Düsseldorf, Düsseldorf, Germany) based on data from a previously published study evaluating the awareness and acceptance of dental implants in the general population [7]. A confidence level of 95% and an acceptable margin of error of 6% were considered for sample size estimation. The minimum sample size required was 251 participants. To compensate for incomplete responses and possible nonresponse bias, an additional 10% was added, resulting in a final sample size of 276 participants.

Convenience sampling was used to recruit participants. Eligible participants were approached consecutively during the study period until the desired sample size was achieved.

Questionnaire design and validation

Data were collected using a structured self-administered questionnaire developed after reviewing previously published literature related to dental implant awareness and acceptance (Appendices). The questionnaire consisted of two sections. The first section included demographic variables, such as age, gender, educational status, monthly family income, and occupational profile. The second section included 20 implant-related questions assessing awareness, knowledge, attitude, willingness, barriers, and sources of information regarding dental implants.

The questionnaire was further categorized into four domains: basic awareness, specific knowledge, attitude, willingness, and barriers to information sources. Before the commencement of the main study, the questionnaire was pilot tested on a small group of participants (n = 30) to assess clarity, readability, and feasibility. The necessary modifications were incorporated based on participant feedback.

Scoring criteria

The questionnaire included three major domains. Basic awareness consisted of four questions with scores ranging from 0 to 4. Specific knowledge included nine questions with scores ranging from 0 to 9. Attitude and willingness were assessed using four questions, with scores ranging from 0 to 8. Higher scores indicate better awareness, greater factual knowledge, and more favorable attitudes toward dental implants. Questions related to barriers and information sources were analyzed descriptively and were not included in the composite score calculation.

Data collection procedure

Participants fulfilling the eligibility criteria were approached individually and informed of the purpose of the study. After obtaining written informed consent, questionnaires were distributed and completed in the presence of the investigator to minimize missing responses and to clarify participant doubts when necessary. Participants were instructed not to discuss their responses with others during questionnaire completion. Completed questionnaires were immediately collected and checked for completeness before inclusion in the final dataset.

Outcome measures

The primary outcome measures included assessment of basic awareness, specific knowledge, attitude and willingness, and the total implant awareness score among the participants. Secondary outcome measures included the identification of major barriers preventing implant acceptance and the evaluation of common sources of information regarding dental implants.

Statistical analysis

Data were analyzed using the Statistical Package for the Social Sciences (SPSS) software version 26.0 (released 2018; IBM Corp., Armonk, USA). The Shapiro-Wilk test was used to assess the normality of data distribution. Descriptive statistics are expressed as mean ± standard deviation for continuous variables and frequency with percentage for categorical variables. An independent t-test was used for comparisons between gender groups, while one-way analysis of variance (ANOVA) was applied for comparisons between education and income groups. Post-hoc pairwise comparisons were performed using Tukey’s honest significant difference test. Pearson’s correlation coefficient was used to assess correlations between score categories. Differences were considered statistically significant at a p-value < 0.05.

## Results

A total of 276 participants were included in this study. The demographic characteristics of the study population are shown in Table 1. Among the participants, 152 (55.1%) were male, and 124 (44.9%) were female. Regarding educational status, 48 (17.4%) participants had no formal education, 92 (33.3%) had primary or secondary education, 98 (35.5%) had completed higher secondary education or graduation, and 38 (13.8%) were postgraduates. Regarding monthly family income, 62 (22.5%) participants belonged to the income group below ₹10,000, 104 (37.7%) earned ₹10,000-25,000, 70 (25.4%) earned ₹25,001-50,000, and 40 (14.5%) earned more than ₹50,000 per month. A total of 184 (66.7%) participants reported having a missing tooth, whereas 92 (33.3%) did not report tooth loss (Table 1).

**Table 1:** Demographic characteristics of the study sample. INR: Indian rupee

Characteristic	Category	n (%)
Gender	Male	152 (55.1)
	Female	124 (44.9)
Education	No formal education	48 (17.4)
	Primary/secondary	92 (33.3)
	Higher secondary/graduate	98 (35.5)
	Postgraduate	38 (13.8)
Monthly family income (INR)	< 10,000	62 (22.5)
	10,000-25,000	104 (37.7)
	25,001-50,000	70 (25.4)
	> 50,000	40 (14.5)
Have a missing tooth	Yes	184 (66.7)
	No	92 (33.3)

A comparison of the questionnaire scores according to gender is presented in Table 2. Female participants demonstrated comparatively higher mean scores than male participants across all score domains. The mean basic awareness score was 2.68 ± 1.08 among females and 2.42 ± 1.15 among males, although the difference was not statistically significant (p = 0.056). Similarly, specific knowledge scores were higher among females (4.21 ± 1.95) than among males (3.85 ± 2.10), but the difference was not statistically significant (p = 0.145). Attitude and willingness scores were also marginally higher among females (3.75 ± 2.05) than among males (3.28 ± 1.98), with borderline significance (p = 0.054). However, the total questionnaire score was significantly higher among female participants (10.64 ± 4.10) than among male participants (9.55 ± 4.35) (p = 0.037), indicating better overall awareness and acceptance of dental implants among females (Table 2).

**Table 2:** Comparison of mean scores by gender (independent t-test). Data are presented as mean ± standard deviation (SD). The independent t‑test was used for intergroup comparison. Degrees of freedom (df) = 274 for all comparisons. Score ranges: Basic awareness (0-4), Specific knowledge (0-9), Attitude and willingness (0-8), Total score (0-21). *p < 0.05 was considered statistically significant.

Score category	Male, n = 152 (55.1%)	Female, n = 124 (44.9%)	t‑value (df = 274)	p‑value
	Mean ± SD	Mean ± SD		
Basic awareness (0-4)	2.42 ± 1.15	2.68 ± 1.08	1.92	0.056
Specific knowledge (0-9)	3.85 ± 2.10	4.21 ± 1.95	1.46	0.145
Attitude and willingness (0-8)	3.28 ± 1.98	3.75 ± 2.05	1.93	0.054
Total score (0-21)	9.55 ± 4.35	10.64 ± 4.10	2.09	0.037*

A comparison of the questionnaire scores according to educational status is presented in Table 3. A progressive increase in the mean scores was observed with increasing educational level. Participants with postgraduate education demonstrated the highest mean scores across all domains, whereas participants without formal education showed the lowest scores. Basic awareness scores increased from 1.85 ± 0.92 among participants without formal education to 3.15 ± 0.85 among postgraduates. Similarly, specific knowledge scores increased from 2.45 ± 1.60 to 5.80 ± 1.75, while attitude and willingness scores increased from 2.10 ± 1.45 to 4.60 ± 1.80. The total questionnaire scores also demonstrated a stepwise increase from 6.40 ± 3.20 among participants without formal education to 13.55 ± 3.80 among postgraduate participants. All differences were statistically significant (p < 0.001), indicating a strong association between higher educational attainment and improved awareness, knowledge, and acceptance of dental implants (Table 3).

**Table 3:** Comparison of mean scores by education level (one-way ANOVA). Data are presented as mean ± SD. One‑way ANOVA was used for comparisons across education levels. Degrees of freedom: between groups = 3, within groups = 272. *p < 0.05 was considered statistically significant. SD: standard deviation; ANOVA: analysis of variance

Education level	No formal education	Primary/secondary	Higher secondary/graduate	Postgraduate	ANOVA F (df=3,272)	p‑value
N (%)	48 (17.4)	92 (33.3)	98 (35.5)	38 (13.8)		
Basic awareness	1.85 ± 0.92	2.30 ± 1.05	2.75 ± 1.10	3.15 ± 0.85	12.45	< 0.001*
Specific knowledge	2.45 ± 1.60	3.50 ± 1.85	4.60 ± 2.00	5.80 ± 1.75	20.67	< 0.001*
Attitude and willingness	2.10 ± 1.45	3.20 ± 1.80	4.05 ± 1.95	4.60 ± 1.80	15.32	< 0.001*
Total score	6.40 ± 3.20	9.00 ± 3.85	11.40 ± 4.20	13.55 ± 3.80	24.18	< 0.001*

Post-hoc pairwise comparisons between education groups using Tukey’s honestly significant difference test are shown in Table 4. Most pairwise comparisons demonstrated statistically significant differences in the awareness, knowledge, attitude, and total scores. Participants without formal education scored significantly lower than all higher educational groups across all domains. Significant differences were also observed between the primary/secondary and higher education groups. A comparison between the higher secondary/graduate and postgraduate groups remained statistically significant for awareness, knowledge, and total score domains, whereas attitude and willingness scores did not show statistical significance (p = 0.06) (Table 4).

**Table 4:** Post-hoc analysis (Tukey's test) for education level. Pairwise comparisons were performed using the Tukey post‑hoc test following a significant one‑way ANOVA. Values shown are t‑values and adjusted p‑values for multiple comparisons. *p < 0.05 was considered statistically significant. vs: versus; ANOVA: analysis of variance

Pairwise comparison	Basic awareness		Specific knowledge		Attitude and willingness		Total score	
	t-value	p-value	t-value	p-value	t-value	p-value	t-value	p-value
No formal education vs. primary/secondary	-2.512	0.013*	-3.412	0.001*	-3.557	<0.001*	-4.249	<0.001*
No formal education vs. higher secondary/graduate	-4.761	<0.001*	-6.784	<0.001*	-5.614	<0.001*	-7.972	<0.001*
No formal education vs. postgraduate	-6.458	<0.001*	-8.955	<0.001*	-6.489	<0.001*	-9.282	<0.001*
Primary/secondary vs. higher secondary/graduate	-2.843	0.005*	-4.085	<0.001*	-2.914	0.004*	-4.109	<0.001*
Primary/secondary vs. postgraduate	-4.792	<0.001*	-6.274	<0.001*	-4.102	<0.001*	-6.185	<0.001*
Higher secondary/graduate vs. postgraduate	-2.018	0.045*	-3.129	0.002*	-1.892	0.06	-2.873	0.005*

A comparison of the mean questionnaire scores according to monthly family income is presented in Table 5. Participants belonging to the higher income groups demonstrated consistently greater awareness, knowledge, and favorable attitudes toward dental implants. Basic awareness scores increased from 2.05 ± 0.95 in the lowest income group (<₹10,000) to 3.10 ± 0.90 in the highest income group (>₹50,000). Specific knowledge scores increased from 2.90 ± 1.70 to 5.60 ± 1.60, while attitude and willingness scores improved from 2.60 ± 1.70 to 4.50 ± 1.75. Total questionnaire scores showed a steady rise from 7.55 ± 3.50 among participants earning less than ₹10,000 to 13.20 ± 3.70 among participants earning more than ₹50,000 per month. All comparisons were statistically significant (p < 0.001), demonstrating a strong positive relationship between socioeconomic status and implant awareness and acceptance (Table 5).

**Table 5:** Comparison of mean scores by monthly family income (one-way ANOVA). Data are presented as mean ± SD. One‑way ANOVA was used for comparisons across monthly family income groups (INR). Degrees of freedom: between groups = 3, within groups = 272. *p < 0.05 was considered statistically significant. SD: standard deviation; ANOVA: analysis of variance

Income group (INR)	< 10,000	10,000-25,000	25,001-50,000	> 50,000	ANOVA F (df=3,272)	p‑value
N (%)	62 (22.4)	104 (37.7)	70 (25.4)	40 (14.5)		
Basic awareness	2.05 ± 0.95	2.40 ± 1.10	2.85 ± 1.05	3.10 ± 0.90	8.94	< 0.001*
Specific knowledge	2.90 ± 1.70	3.80 ± 1.95	4.70 ± 1.85	5.60 ± 1.60	16.23	< 0.001*
Attitude and willingness	2.60 ± 1.70	3.40 ± 1.90	4.00 ± 1.85	4.50 ± 1.75	10.45	< 0.001*
Total score	7.55 ± 3.50	9.60 ± 4.00	11.55 ± 4.10	13.20 ± 3.70	18.92	< 0.001*

The post-hoc pairwise comparisons of income groups using Tukey’s test are presented in Table 6. Significant differences were observed between most income categories across all score domains. Participants in the lowest income group demonstrated significantly lower awareness, knowledge, attitude, and total scores than those in the higher income groups. Comparisons between the middle- and higher-income groups were also statistically significant in most domains. However, comparisons between the ₹25,001-50,000 and >₹50,000 income groups did not demonstrate statistically significant differences in basic awareness and attitude scores (Table 6).

**Table 6:** Post-hoc analysis (Tukey's test) for income level. Pairwise comparisons were performed using the Tukey post‑hoc test following a significant one‑way ANOVA. Values shown are t‑values and adjusted p‑values for multiple comparisons. *p < 0.05 was considered statistically significant. Income groups are in Indian Rupees (INR) per month. vs: versus; ANOVA: analysis of variance

Pairwise comparison	Basic awareness		Specific knowledge		Attitude and willingness		Total score	
	t-value	p-value	t-value	p-value	t-value	p-value	t-value	p-value
<10,000 vs. 10,000-25,000	-2.115	0.035*	-3.162	0.002*	-2.684	0.008*	-3.458	<0.001*
<10,000 vs. 25,001-50,000	-4.228	<0.001*	-5.871	<0.001*	-4.571	<0.001*	-6.046	<0.001*
<10,000 vs. >50,000	-5.376	<0.001*	-7.494	<0.001*	-5.819	<0.001*	-7.690	<0.001*
10,000-25,000 vs 25,001-50,000	-2.467	0.014*	-3.026	0.003*	-2.175	0.031*	-3.107	0.002*
10,000-25,000 vs >50,000	-3.822	<0.001*	-5.108	<0.001*	-3.714	<0.001*	-5.111	<0.001*
25,001-50,000 vs >50,000	-1.214	0.226	-2.231	0.027*	-1.562	0.12	-2.162	0.033*

The correlation analysis revealed significant positive associations among the three domains of implant awareness and attitude. Basic awareness showed a strong positive correlation with specific knowledge (r = 0.58). Attitude and willingness also demonstrated a strong positive correlation with specific knowledge (r = 0.62). Additionally, attitude and willingness was moderately correlated with basic awareness (r = 0.49). All correlation coefficients were statistically significant, indicating that higher basic awareness is associated with better specific knowledge and more favorable attitudes toward dental implants (Table 7).

**Table 7:** Pearson's correlation between score categories. *p < 0.001 (two‑tailed) significant for all correlations. Pearson (r) values: <0.10 negligible, 0.10-0.29 weak, 0.30-0.49 moderate, 0.50-0.69 strong, 0.70-1.00 very strong to perfect.

Paired variables	r	p-value
Basic awareness vs. specific knowledge	0.58	< 0.001*
Attitude and willingness vs. specific knowledge	0.62	< 0.001*
Attitude and willingness vs. basic awareness	0.49	< 0.001*

## Discussion

Dental implants have become one of the most predictable and successful treatment modalities for the replacement of missing teeth because of their superior functional efficiency, esthetics, and long-term prognosis [4]. However, the successful implementation of implant therapy largely depends on patient awareness, knowledge, socioeconomic status, and willingness to undergo treatment. The present study evaluated the awareness, knowledge, attitude, and acceptance of dental implants among the North Maharashtra population and demonstrated that educational status and monthly family income significantly influenced implant-related awareness and acceptance.

In the present study, overall awareness regarding dental implants was moderate, with considerable variation among the different demographic groups. Female participants demonstrated comparatively higher mean scores in all domains, and the total score was significantly greater among females than among males. Similar findings were reported by Siddique et al. [7], who observed greater awareness and acceptance among female participants, possibly because females are generally more concerned about oral aesthetics and appearance. Previous studies conducted by Pommer et al. [8] and Tepper et al. [9] also reported slightly higher awareness among women, although the sex-based differences were not consistently statistically significant. The higher scores among females in the present study may reflect increased cosmetic awareness and greater interest in oral healthcare among women.

Educational status had a strong positive association with implant awareness, knowledge, and attitude. Participants with postgraduate education had the highest scores, whereas those without formal education had the lowest scores across all domains. These findings are in agreement with those of Siddique et al. [7], Padhye et al. [10], and Khalifa et al. [11], who reported that higher educational attainment significantly improves awareness regarding advanced dental treatment modalities. Educated individuals are more likely to access healthcare information through dentists, social media, and Internet sources, thereby increasing their understanding of implant therapy. Better education may also improve comprehension of implant longevity, maintenance, and success rates, leading to more favorable treatment attitudes.

Monthly family income was another significant determinant of implant awareness and acceptance in the present study. Participants belonging to the higher income groups demonstrated significantly greater awareness, knowledge, and willingness toward implant therapy compared to those in the lower income groups. These findings are consistent with those of studies by Suprakash et al. [12] and Kranjcic et al. [13], who reported that financial affordability is one of the strongest predictors of acceptance of dental implants. Implant treatment is often perceived as expensive, especially in developing countries, such as India, where insurance coverage for implant therapy is limited. In the present study, cost-related concerns emerged as a major barrier affecting implant acceptance, indicating that economic constraints remain a major obstacle to the widespread utilization of implant therapy.

The present study also demonstrated strong positive correlations between the awareness, knowledge, and attitude scores. Specific knowledge showed the strongest correlation with the total awareness score, suggesting that factual understanding of implant treatment plays a critical role in patient acceptance. Similar observations were reported by Zimmer et al. [14], who concluded that patients with accurate information regarding implant procedures and prognosis were more likely to choose implants over conventional prostheses. This highlights the importance of patient education and effective communication between dental professionals.

Another important finding of this study was the role of information sources in shaping implant awareness. Previous studies suggest that dentists remain the most reliable and influential source of implant-related information, followed by social media and family members. Previous studies emphasized the importance of professional counseling in improving patient understanding and reducing misconceptions regarding implant therapy [15,16]. In the present study, participants with greater knowledge also exhibited more favorable attitudes, indicating that targeted awareness programs and patient education initiatives may significantly improve treatment acceptance.

From a clinical perspective, the findings of the present study emphasize the need for improved public education on dental implants, particularly among individuals with lower educational and socioeconomic backgrounds. Dental professionals should provide comprehensive counseling regarding implant indications, benefits, longevity, maintenance requirements, and treatment outcomes. Community-based awareness programs, educational campaigns, and incorporation of implant education into routine dental consultations may help bridge the existing knowledge gaps. Additionally, improving affordability through insurance support or subsidized treatment programs may enhance the acceptance of implant therapy among economically disadvantaged populations.

Despite these important findings, the present study had several limitations. This study employed a convenience sampling method, which may limit the generalizability of the findings to the entire population. Being a questionnaire-based cross-sectional study, participant responses may have been influenced by recall or social desirability bias. This study was conducted in a single institutional setting associated with North Maharashtra; therefore, regional variations in awareness may not have been fully represented. Furthermore, the clinical evaluation of the participants and assessment of actual treatment uptake were not performed. Future multicenter studies with larger sample sizes and longitudinal follow-ups are recommended to better evaluate implant awareness and treatment acceptance across broader populations.

## Conclusions

The present study demonstrated that awareness, knowledge, and acceptance of dental implants among the North Maharashtra population were moderate and significantly influenced by educational status and monthly family income. Participants with higher education and better socioeconomic status exhibited greater awareness, improved factual knowledge, and more favorable attitudes toward implant therapy. Females showed slightly higher overall awareness scores than males. The cost and lack of adequate information remain major barriers to implant acceptance. These findings highlight the need for targeted public education programs, improved patient counseling, and increased accessibility of implant therapy to enhance awareness and acceptance of dental implants in the general population.

## Appendices

**Table 8:** Questionnaire for the study. All questions were single-select, closed-ended. Demographic variables (part 1) were recorded as categorical groups. The questionnaire was pretested for clarity and content validity on 30 subjects from the target population before the main study.

Part 1-Demographic questions								
S. no.	Question	Response						
1	Age (years)	8-30	31-45	46-60		>60		
2	Gender	Male	Female					
3	Education	No formal education	Primary/secondary		Higher secondary/graduate			Postgraduate
4	Economic status (income per month)	< 10,000 INR	10,000-25,000 INR		25,001-50,000 INR			>50,000 INR
5	Work profile	Non-working	Working		Retired			
Part 2-implant-related questions								
S. no.	Question						Response	
Q1	Do you have a missing tooth? Yes/No							
Q2	Do you think replacement of missing teeth is important? Yes/No							
Q3	Are you aware that various treatment options are available for the replacement of missing teeth? Yes /No							
Q4	Have you ever heard about dental implants? Yes/No							
Q5	If you have a missing tooth/teeth, what treatment option will you opt for? Removable/Fixed Dental Implant/Not sure							
Q6	Have you ever had a dental implant? Yes/No							
Q7	According to you, what is a dental implant? An artificial tooth root placed in jawbone/A screw that holds a false tooth/A fake tooth that sits on the gums/Don’t know							
Q8	Where do you think the implant is placed? Gums/Bone/Tooth/Don’t know							
Q9	What material is a dental implant made of? Ceramic/Porcelain/Titanium/Don’t know							
Q10	What part of the tooth does a dental implant replace? Crown/Root/Both/None/Don’t know							
Q11	How long do you think a dental implant lasts? <5 years/5-10 years/>10 years							
Q12	Do you know whether your dentist provides implants? Yes/No							
Q13	Who is most qualified to place dental implants? General dentist/Specialist (oral surgeon, periodontist, etc.)/Both/Don’t know							
Q14	Are you aware that dental implant treatment is covered by insurance? Yes/No							
Q15	Do you think implants need special care? Yes/No/Maybe							
Q16	Main source of information about dental implants? Social media/Dentist/Family and Friends/Newspaper/Other sources (Internet, TV)							
Q17	Would you like to know more about dental implants? Yes/No/Not sure							
Q18	Main reason for not choosing the implant? High cost/Fear of surgery/Fear of foreign material/Complications/Time-consuming/Lack of information							
Q19	Would you be willing to travel to a nearby city if implants are not available locally? Yes/No/Not sure							
Q20	Why do implants fail? Poor oral hygiene/Smoking/Inadequate bone quality/Surgeon’s inexperience/Implant material defect/Don’t know							
